# Inactivated vaccine effectiveness against symptomatic COVID-19 in Fujian, China during the Omicron BA.2 outbreak

**DOI:** 10.3389/fpubh.2023.1269194

**Published:** 2023-12-14

**Authors:** Wenjing Ye, Kangguo Li, Zeyu Zhao, Shenggen Wu, Huimin Qu, Yichao Guo, Buasiyamu Abudunaibi, Wu Chen, Shaojian Cai, Cailin Chen, Jiawei Lin, Zhonghang Xie, Meirong Zhan, Jianming Ou, Yanqin Deng, Tianmu Chen, Kuicheng Zheng

**Affiliations:** ^1^Institute of Emergency Response and Epidemic Management, Fujian Provincial Center for Disease Control and Prevention, Fuzhou, China; ^2^State Key Laboratory of Vaccines for Infectious Diseases, Xiang an Biomedicine Laboratory, State Key Laboratory of Molecular Vaccinology and Molecular Diagnostics, National Innovation Platform for Industry-Education Integration in Vaccine Research, School of Public Health, Xiamen University, Xiamen, China

**Keywords:** COVID-19, SARS-CoV-2, Omicron BA.2, vaccine effectiveness (VE), inactivated vaccine

## Abstract

**Objective:**

More than 90% of the Chinese population have completed 2 doses of inactivated COVID-19 vaccines in Mainland China. However, after China government abandoned strict control measures, many breakthrough infections appeared, and vaccine effectiveness against Omicron BA.2 infection was uncertain. This study aims to investigate the real-world effectiveness of widely used inactivated vaccines during the wave of Omicron variants.

**Methods:**

Test-negative case-control study was conducted in this study to analyze the vaccine effectiveness against symptomatic disease caused by the Omicron variant (BA.2) in Fujian, China. Conditional logistic regression was selected to estimate the vaccine effectiveness.

**Results:**

The study found the vaccine effectiveness against symptomatic COVID-19 is 32.46% (95% CI, 8.08% to 50.37%) at 2 to 8 weeks, and 27.05% (95% CI, 1.23% to 46.12%) at 12 to 24 weeks after receiving booster doses of the inactivated vaccine. Notably, the 3–17 years group had higher vaccine effectiveness after 2 doses than the 18–64 years and over 65 years groups who received booster doses.

**Conclusion:**

Inactivated vaccines alone may not offer sufficient protection for all age groups before the summer of 2022. To enhance protection, other types of vaccines or bivalent vaccines should be considered.

## Introduction

In December 2022, the Chinese government abandoned its “dynamic zero-COVID” policy and adopted a new measure that sought to live with the virus. The new measures included relaxed restrictions on international flights and discontinuation of mass nucleic acid testing. However, these changes led to a significant increase in new infections, higher morbidity and mortality rates, and severe clinical symptoms, particularly among older adult patients in Mainland China. This underscores the urgent need for effective measures to control the spread of COVID-19 and mitigate its impact on vulnerable populations. Moreover, a modeling study indicated that cumulative infection rates in places to which Beijing belongs have more than 90% (1) which were inconsistent with high vaccine coverage rates in Mainland China. There were three types of COVID-19 vaccines including inactivated vaccine, adenovirus vector vaccine, and recombinant protein vaccine. Among these, more than 90% of Chinese citizens has completed 2 doses of inactivated COVID-19 vaccines produced by Sinovac Biotech Ltd. and Sinopharm Group Co. Ltd. (2). Which developed based on wild-type SARS-CoV-2 in 2020 and the effectiveness decreased remarkably in the Delta variant wave. In Omicron BA.2 variant wave, Wan et al. have previously reported that the vaccine effectiveness against infection of the CoronaVac vaccine was 19.8% after the booster dose and not observed after 2 doses (3).

Unquestionably, inactivated COVID-19 vaccines are inefficient against infection and symptoms. However, understanding when inactivated vaccines lose their effectiveness still holds significant reference value for future vaccine development and the response to COVID-19.

## Methods

### Study design

To estimate the effectiveness against symptomatic COVID-19 caused by the Omicron BA.2 variant of inactivated vaccine with either CoronaVac (Sinovac Biotech Ltd.) and COVILO (Sinopharm Group Co. Ltd.) vaccines, as compared to varied age groups, we used a test-negative case-control design in the contact population of Fujian, China.

### Data source

COVID-19 laboratory testing, vaccination status and demographic characteristics were provided by the Fujian Provincial Center for Disease Control and Prevention, which was extracted from the national surveillance system for infectious diseases and vaccination system in China. These systems were developed and supervised by National Health Commission of the People's Republic of China and stored all patient information.

### Study population

The study was conducted in Fujian province during the outbreaks of the Omicron BA.2 variant from March 13 to April 24, 2022. The inclusion criteria consisted of participants included symptomatic cases aged over 3 years who had a confirmed diagnosis of COVID-19. The symptomatic case was defined as individuals who tested positive for the Omicron BA.2 variant of COVID-19 and reported symptoms consistent with the disease according to the 9th edition of COVID-19 protocols for diagnosis and treatment in the People's Republic of China. These symptoms include fever, cough, fatigue, stuffy and runny nose, sore throat, shortness of breath, muscle pain, diarrhea, and impaired sense of smell and taste. We adopted this definition of symptomatic cases to focus specifically on the effects of the vaccines on the development of symptoms caused by the Omicron BA.2 variant. For comparison, we included controls who were contacts of infected individuals but tested negative for the Omicron BA.2 variant or tested positive but did not exhibit any symptoms.

### Vaccination status

To properly assess the effectiveness of the vaccines, we needed to accurately determine the vaccination status of each participant in the study. To do this, we thoroughly reviewed the records provided by the national vaccination system, which included verifying the vaccination dates, the type of vaccine administered, and any potential errors or inconsistencies in the data across vaccines administered in a regular institution in mainland China. In this analysis, we excluded individuals who had received adenoviral or mRNA or protein subunit vaccines, as our focus was on the effects of inactivated vaccines on developing COVID-19 symptoms caused by the Omicron BA.2 variant. Additionally, we included individuals who had not received any doses as part of our study population.

### Statistical analysis

Our analysis used conditional logistic regression to estimate the odds ratios (OR) for developing symptomatic COVID-19 associated with vaccination with either CoronaVac or COVILO. The effectiveness of the vaccines was then assessed by calculating (1 – OR) × 100%, where OR represents the odds ratio for developing symptoms among vaccinated individuals compared to unvaccinated individuals. We included these variables in our logistic regression model to control for potential bias such as age, gender, and underlying health conditions.

In addition to the overall analysis, we also conducted stratified analyses by age group to assess some potential differences in vaccine effectiveness by age, which allowed us to determine whether the vaccines were more or less effective in different age groups, which could inform vaccination strategies and prioritize certain age groups for vaccination. All statistical analysis and generated figures were conducted using R version 4.2.1 (R Core Team, Vienna, Austria), and the source code is available under the GNU General Public License version 3 at the GitHub repository (https://github.com/xmusphlkg/inactivated_vaccine_effectiveness).

## Results

We collected data on 98,053 contacts of infections for 40 days (from March 13 to April 24, 2022) at the time of the Omicron BA.2 outbreak in Fujian province, China, and a case-control study design was deployed to analyze the actual effectiveness of inactivated vaccines, the actual proportion of asymptomatic infections and provide some suggestions for the adjustment of the COVID-19 vaccination strategy in the mainland China.

**Figure 1:**
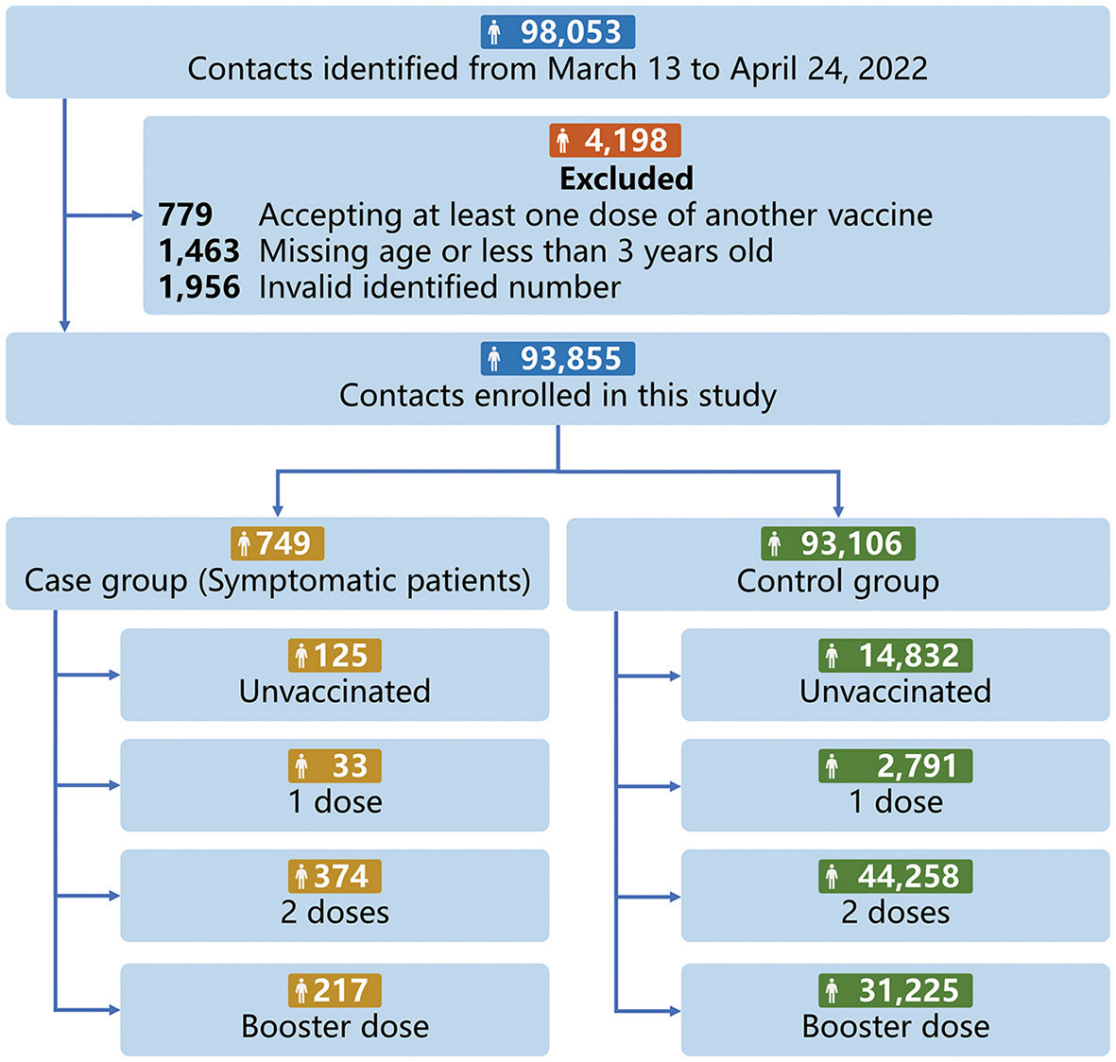
Participants selection progress for investing inactivated vaccine effectiveness.

The vaccine status database of 98,053 contacts (caused by 3,578 infections) in the outbreak were extracted from the local health system according to the valid ID number. Which recorded the dose, date, manufacturer, and clinical of each legal vaccine accepted in mainland China; 93,855 contacts were enrolled in the analysis of inactivated vaccine effectiveness (Figure 1). The most common reason for exclusion was the invalid identified number (*n* = 1,956), and 779 contacts were excluded for accepting at least one dose of another vaccine, including Ad5-nCoV-S, recombinant vaccine. For vaccine status, the last vaccine dose received after 14 days is considered valid. A total of 44,632 contacts had completed 2 vaccine doses, and 31,442 contacts had received the booster before this outbreak. The median time between the last vaccine date and expose date was 126 days (IQR: 79–210 days).

Symptomatic patients with SARS-CoV-2 were identified according to the 9th edition of COVID-19 protocols for diagnosis and treatment (4). Of 93,855 contacts of Omicron infections, 749 contacts finally developed as symptomatic patients of the Omicron variant and were selected as the case group in this study (Supplementary Table 1). In total of 624 patients received at least 1 dose of vaccine, and 217 patients received the booster dose. Symptomatic breakthrough infection was defined as SARS-CoV-2 infection with symptoms 14 days after the second dose of vaccine (5). And compared to unvaccinated patients, the median age of breakthrough patients tended to be higher (31 vs. 34 years) (Supplementary Table 2). Among 624 breakthrough infections, 103 (16.51%) patients received the BBIBP-CorV vaccine, 195 (31.25%) patients received the CoronaVac vaccine, and 331 (53.04%) received the mixed inactivated vaccine produced by different manufacturers. It is important to note that the mixed inactivated vaccine refers to a combination of inactivated vaccines from different manufacturers, and not a heterologous prime-boost approach involving other platforms. For the control group, we selected 93,106 contacts who were exposed in the outbreak between March 13 to April 24, 2022, but did not develop symptomatic infections. A total of 44,258 contacts (47.54%) received 2 dose inactivated vaccine and 31,225 contacts (33.54%) received booster dose (Supplementary Table 2).

**Figure 2:**
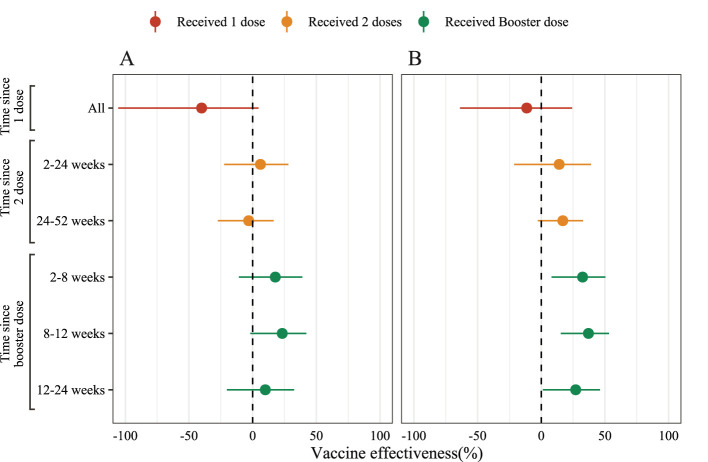
The estimated effectiveness of inactivated COVID-19 vaccines against symptomatic SARS-CoV-2 infections caused by Omicron BA.2. Unadjusted **(A)** and adjusted **(B)** logistic conditional model by age group (3–17 years, 18–64 years and older than 65 years) and gender (male and female) to estimated vaccine effectiveness by various intervals.

**Figure 3:**
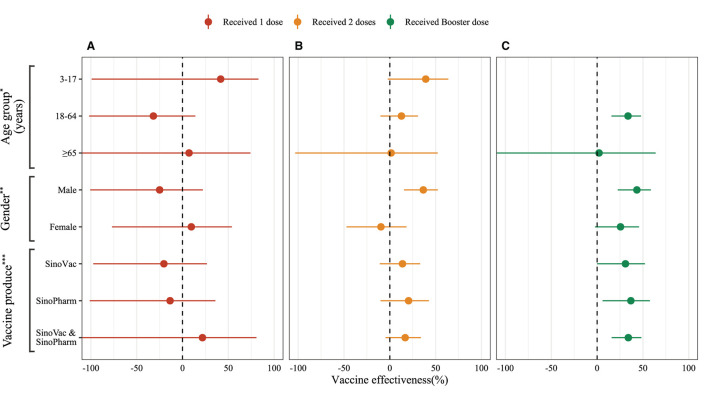
Adjusted vaccine effectiveness of inactivated COVID-19 vaccines by various vaccine doses against symptomatic SARS-CoV-2 infections caused by Omicron BA.2. **(A)** 1 dose inactivated vaccine effectiveness. **(B)** 2 doses inactivated vaccine effectiveness. **(C)** Booster dose inactivated vaccine effectiveness. ^*^Model adjusted by gender (male and female); ^**^Model adjusted by age groups (3–17 years, 18–64 years, and more than 65 years); ^***^Model adjusted by age groups and gender.

In this analysis, we used conditional logistic regression and adjusted by gender and age group (3–17 years, 18–64 years, and over 65 years). The vaccine effectiveness estimated using (1 – *OR*_*adjusted*_) × 100%. All statistical analyses were performed in R 4.2.1 (6), and the source code is available on GitHub (https://github.com/xmusphlkg/inactivated_vaccine_effectiveness). Overall, the estimated effectiveness of the booster dose was 32.46% (95%CI, 8.09% to 50.37%) between week 2 and week 8, and decreased to 27.05% (95%CI, 1.23% to 46.12%) between week 12 and week 24. However, whether adjusted by age groups and gender or not, we did not observe vaccine effectiveness after 1 dose of inactivated vaccine and 24 weeks after 2 doses of vaccine (Figure 2, Supplementary Table 3), which indicated that all un-fully vaccinated persons and last vaccination was given more than 24 weeks ago should start further vaccination as soon as possible. Figure 3 shows the estimated vaccine effectiveness against symptomatic COVID-19 for separate groups. Vaccine effectiveness against symptomatic COVID-19 in children (3–17 years) was higher than adults (18–64 years), despite not receiving the booster dose; in older adults (over 65 years), no vaccine effectiveness against symptomatic COVID-19 was observed regardless of the vaccine dose. Among adults aged 18–64 years, the vaccine effectiveness against symptomatic COVID-19 after accepting 2 dose vaccine was 12.68% (95%CI, −10.21% to 30.82%), and 33.76% (95%CI, 15.75% to 47.91%) for booster dose; among children aged 3–17 years, the effectiveness of 2 doses was 39.23% (95%CI, −2.15% to 63.85%) (Supplementary Table 4), slightly higher than previous reports (7). No major differences were found across vaccine manufacturers, and the mixing vaccine strategy within inactivated vaccines failed to improve the vaccine effectiveness in the Omicron BA.2 wave.

## Discussion

The vaccine effectiveness of the inactivated vaccine against symptomatic COVID-19 appeared to be lower than reported in Brazil and Colombia (8, 9), and close to Hui Yang et al. reported that full vaccination does not show vaccine effectiveness against server Omicron BF.7 variant in Beijing, China (10). There are several potential factors that may contribute to this disparity in vaccine effectiveness. Firstly, the prevalence of different SARS-CoV-2 variants in each country could play a significant role. The Omicron variants have demonstrated increased susceptibility to immune evasion and breakthrough infections when compared to the Delta variant and the wild type that predominated before January 2022 (11). Secondly, variations in the accepted last vaccine date and vaccination strategies, including the scheduling and spacing of doses, could have an impact on the overall effectiveness. Differences in these strategies across countries could contribute to the observed disparities in vaccine effectiveness. The median time between the last vaccine date and exposure date was 126 days, which is close to the recommended vaccine dosing interval of 180 days (12). This suggests that despite the vaccine coverage in Mainland China nearing 100%, the rapid decline in vaccine effectiveness means that most individuals are no longer protected. Furthermore, the consistent implementation of public health and social measures (PHSMs) in mainland China, such as social distancing, mask-wearing, and travel restrictions, plays a crucial role in reducing the transmission of the virus and overall physical contacts (13, 14). These comprehensive containment measures have the potential to significantly reduce the transmissibility of variants. For instance, the proper use of masks can diminish droplet transmission and decrease infections by 47% (15). Similarly, maintaining social distance can limit interpersonal contact and reduce transmission by 12% (16), lowering the risk of infection and transmission. It is important to note that these factors may contribute to an overestimation of vaccine effectiveness, as PHSMs can also reduce the risk of infection among unvaccinated individuals.

The inactivated booster vaccine demonstrates certain advantages compared to full vaccination, even with a faster decline in vaccine efficacy. At 12–24 weeks, it still maintains 27.05% effectiveness (95%CI, 1.22% to 46.12%), which is higher than the 16.93% effectiveness (95%CI, −2.70% to 32.80%) of full vaccination (Supplementary Table 3). Additionally, another larger-scale cohort study revealed that the adjusted effectiveness of the inactivated booster vaccine against Omicron BA.5 infection was 35.5% (95%CI, 2.0 to 57.5%) compared to the two-dose inactivated vaccine, and no protective effect was observed in individuals aged 40 and above (17). This could be attributed to the fact that antibody levels peak around 4–5 weeks after vaccination and subsequently waning over time (18, 19), particularly among older adults and those with chronic inflammation (20). Additionally, most participants receiving the third dose had an interval of 8–24 weeks between last vaccination and exposure, while the subjects receiving the second dose had an interval predominantly between 24–52 weeks (Supplementary Table 2, Supplementary Figure 1). This suggests that the booster dose group has an initial advantage, meaning their antibody decline is less pronounced.

Compared to adults, children aged 3–17 years demonstrate higher vaccine effectiveness against symptomatic COVID-19 when administered inactivated vaccines at the same dosage. This finding is consistent with the efficacy of mRNA vaccines (21) and is likely attributed to unique characteristics of the pediatric immune system. The immune system of children, due to its relatively recent development, possesses a heightened capacity to generate robust and sustained immune responses to SARS-CoV-2 antigen (22). While antibodies are not the only indication of vaccine effectiveness, higher antibody levels decrease the risk of infection and death (20, 23, 24).

Our study has several limitations that need to be acknowledged. Firstly, it is particularly valuable to assess the inactivated vaccine effectiveness against hospitalization, severe COVID-19, and mortality during epidemic periods. However, since our study only reported one case of severe COVID-19 and did not record detailed clinical data, we cannot extend our findings on vaccine effectiveness to more severe outcomes of COVID-19. Secondly, we did not consider the problem of reinfection in our study. Due to the “dynamic zero-COVID” policy adopted in Mainland China before November 2022, only a small percentage of residents had been previously infected with SARS-CoV-2. Therefore, there is a possibility that individual cases of reinfection may have been overlooked, although we believe this has minimal impact on the overall results of our study. Thirdly, our study was unable to assess the effectiveness of other vaccine types, such as adenovirus vector vaccines or mRNA vaccines, as their market share in Mainland China is relatively low, making it challenging to collect enough samples. Finally, it is widely acknowledged that vaccine effectiveness was waning over time (18, 19). However, due to variations in public vaccinated willingness, the distribution of interval between the last vaccine dose and exposure among individuals was concentrated within a specific range (Supplementary Figure 1). Consequently, we could not explore vaccine persistence because vaccination dates and epidemic size were uncontrollable to us.

The lower effectiveness of inactivation against symptomatic COVID-19 may challenge medical resources in the Omicron BA.2 wave, especially in adults over 65. It is time to develop a novel vaccine against the Omicron variant and approve it for widespread use in all age stages. Fortunately, Chinese authorities have recently approved new COVID-19 vaccines for emergency use and plan nationwide 4^th^ dose in response to lower vaccine effectiveness against symptomatic COVID-19 (25).

## Data availability statement

The datasets presented in this study can be found in online repositories. The names of the repository/repositories and accession number(s) can be found in the article/Supplementary material.

## Ethics statement

The studies involving humans were approved by the Institutional Ethics Committee of the Fujian Provincial Center for Disease Control and Prevention (CDC), Fuzhou, China. The studies were conducted in accordance with the local legislation and institutional requirements. Written informed consent for participation in this study was provided by the participants' legal guardians/next of kin.

## Author contributions

WY: Validation, Writing – original draft, Writing – review & editing. KL: Methodology, Software, Writing – original draft, Writing – review & editing. ZZ: Methodology, Writing – review & editing. SW: Writing – review & editing. HQ: Writing – original draft, Writing – review & editing. YG: Investigation, Writing – review & editing. BA: Validation, Writing – original draft, Writing – review & editing. WC: Investigation, Writing – review & editing. SC: Investigation, Writing – review & editing. CC: Investigation, Writing – review & editing. JL: Investigation, Writing – review & editing. ZX: Investigation, Writing – review & editing. MZ: Investigation, Writing – review & editing. JO: Conceptualization, Investigation, Writing – review & editing. YD: Conceptualization, Investigation, Writing – review & editing. TC: Conceptualization, Software, Writing – original draft. KZ: Conceptualization, Investigation, Writing – review & editing.
