# Supplementary material for: Inactivated vaccine effectiveness against symptomatic COVID-19 in Fujian, China during the Omicron BA.2 outbreak
Source: Front Public Health. 2023 Dec 14;11:1269194. doi: 10.3389/fpubh.2023.1269194 (PMC10757624; doi:10.3389/fpubh.2023.1269194)
Supplement: Supplementary file 1 [file Table_1.DOCX]

Supplementary Material

Inactivated vaccine effectiveness against symptomatic COVID-19 in Fujian, China during the Omicron BA.2 outbreak

**Supplementary Table 1**

**Demographic characteristics of participants in Omicron BA.2 outbreaks.**

| **Characteristics** | **No.(%)** |  |
| --- | --- | --- |
|  | **Cases** | **Controls** |
|  | **(N=749)** | **(N=93,106)** |
| Age, median (IQR), yr | 34(23-46) | 33(23-45) |
| Age, yr |  |  |
| 3-17 | 108(14.4) | 14,604(15.7) |
| 18-64 | 599(80.0) | 74,623(80.1) |
| 65- | 42(5.6) | 3,879(4.2) |
| Gender |  |  |
| Female | 355(47.4) | 49,649(53.3) |
| Male | 394(52.6) | 40,430(43.4) |
| Missing | 0(0.0) | 3,027(3.3) |
| Vaccine dose |  |  |
| 0 | 125(16.7) | 14,832(15.9) |
| 1 | 33(4.4) | 2,791(3.0) |
| 2 | 374(49.9) | 44,258(47.5) |
| 3 | 217(29.0) | 31,225(33.5) |
| Vaccine manufacturer |  |  |
| Sinopharm | 103(13.8) | 13,458(14.5) |
| Sinovac | 195(26.0) | 22,855(24.5) |
| Sinovac and Sinopharm | 331(44.2) | 42,640(45.8) |
| Missing | 120(16.0) | 14,153(15.2) |
| Last vaccine date, weeks |  |  |
| Unvaccinated | 125(16.7) | 14,832(15.9) |
| 2-4 | 28(3.7) | 3,793(4.1) |
| 4-8 | 41(5.5) | 6,538(7.0) |
| 8-12 | 94(12.6) | 13,243(14.2) |
| 12-24 | 157(21.0) | 20,839(22.4) |
| 24-52 | 303(40.5) | 33,722(36.2) |
| >52 | 1(0.1) | 139(0.1) |

**Supplementary Table 2**

**Demographic characteristics of symptomatic patients in Omicron BA.2 outbreaks.**

| **Characteristics** | **No.(%)** |  |  |  |
| --- | --- | --- | --- | --- |
|  | **Unvaccinated cases** | **1 dose cases** | **2 doses cases** | **Booster dose cases** |
|  | **(N=125)** | **(N=33)** | **(N=374)** | **(N=217)** |
| Age, median (IQR), yr | 31(22-46) | 32(24-40) | 31(16-44) | 38(32-48) |
| Age, yr |  |  |  |  |
| 1-17 | 17 (13.6) | 3 (9.1) | 88 (23.5) | 0 (0.0) |
| 18-64 | 97 (77.6) | 27 (81.8) | 264 (70.6) | 211 (97.2) |
| 65- | 11 (8.8) | 3 (9.1) | 22 (5.9) | 6 (2.8) |
| Gender |  |  |  |  |
| Female | 58 (46.4) | 10 (30.3) | 207 (55.3) | 119 (54.8) |
| Male | 67 (53.6) | 23 (69.7) | 167 (44.7) | 98 (45.2) |
| Vaccine manufacturer |  |  |  |  |
| Sinopharm | 1 (0.8) | 13 (39.4) | 58 (15.5) | 31 (14.3) |
| Sinovac | 4 (3.2) | 18 (54.5) | 136 (36.4) | 37 (17.1) |
| Sinovac and Sinopharm | 0 (0.0) | 2 (6.1) | 180 (48.1) | 149 (68.7) |
| Missing | 120 (96.0) | 0 (0.0) | 0 (0.0) | 0 (0.0) |
| Last vaccine date, weeks |  |  |  |  |
| Unvaccinated | 125(100.0) | 0(0.0) | 0(0.0) | 0(0.0) |
| 2-4 | 0(0.0) | 1(3.0) | 0(0.0) | 27(12.4) |
| 4-8 | 0(0.0) | 1(3.0) | 1(0.3) | 39(18.0) |
| 8-12 | 0(0.0) | 2(6.1) | 14(3.7) | 78(35.9) |
| 12-24 | 0(0.0) | 2(6.1) | 82(21.9) | 73(33.6) |
| 24-52 | 0(0.0) | 26(78.8) | 277(74.1) | 0(0.0) |
| >52 | 0(0.0) | 1(3.0) | 0(0.0) | 0(0.0) |

**Supplementary Table 3**

**Effectiveness of the inactivated vaccine against COVID-19 symptoms caused by the Omicron BA.2 variant.**

| Receipted Vaccine Dose | Group | Odds Ratio(95%CI) | Adjusted^*^ |
| --- | --- | --- | --- |
| 1 dose | All | 1.11(0.76-1.64) | adjusted |
| 2 doses | 2-24 weeks | 0.86(0.61-1.21) | adjusted |
|  | 24-52 weeks | 0.83(0.67-1.03) | adjusted |
| Booster dose | 2-8 weeks | 0.68(0.5-0.92) | adjusted |
|  | 8-12 weeks | 0.63(0.47-0.85) | adjusted |
|  | 12-24 weeks | 0.73(0.54-0.99) | adjusted |
| 1 dose | All | 1.4(0.95-2.06) | unadjusted |
| 2 doses | 2-24 weeks | 0.94(0.72-1.22) | unadjusted |
|  | 24-52 weeks | 1.03(0.83-1.27) | unadjusted |
| Booster dose | 2-8 weeks | 0.82(0.61-1.11) | unadjusted |
|  | 8-12 weeks | 0.77(0.58-1.02) | unadjusted |
|  | 12-24 weeks | 0.9(0.67-1.2) | unadjusted |

^*^Conditional logistic regression model adjusted by age group (3-17 years, 18-64 years and over 65 years) and gender.

**Supplementary Table 4**

**Subgroup vaccine effectiveness of the inactivated vaccine against COVID-19 symptoms caused by the Omicron BA.2 variant.**

| Group | Receipted Vaccine Dose | Odds Ratio(95%CI) | Adjusted^*^ |
| --- | --- | --- | --- |
| 3-17 years | 1 dose | 0.58(0.17-1.99) | Gender |
|  | 2 doses | 0.61(0.36-1.02) | Gender |
|  | Booster dose | / | Gender |
| 18-64 years | 1 dose | 1.32(0.86-2.02) | Gender |
|  | 2 doses | 0.87(0.69-1.1) | Gender |
|  | Booster dose | 0.66(0.52-0.84) | Gender |
| 65- years | 1 dose | 0.93(0.26-3.33) | Gender |
|  | 2 doses | 0.99(0.48-2.03) | Gender |
|  | Booster dose | 0.98(0.36-2.65) | Gender |
| Male | 1 dose | 1.25(0.78-2.01) | Age group |
|  | 2 doses | 0.63(0.48-0.85) | Age group |
|  | Booster dose | 0.57(0.41-0.77) | Age group |
| Female | 1 dose | 0.9(0.46-1.77) | Age group |
|  | 2 doses | 1.1(0.82-1.47) | Age group |
|  | Booster dose | 0.74(0.54-1.02) | Age group |
| SinoVac | 1 dose | 1.2(0.73-1.97) | Age group & Gender |
|  | 2 doses | 0.86(0.67-1.11) | Age group & Gender |
|  | Booster dose | 0.69(0.48-1) | Age group & Gender |
| SinoPharm | 1 dose | 1.14(0.64-2.01) | Age group & Gender |
|  | 2 doses | 0.8(0.57-1.1) | Age group & Gender |
|  | Booster dose | 0.63(0.42-0.94) | Age group & Gender |
| SinoVac & SinoPharm | 1 dose | 0.78(0.19-3.17) | Age group & Gender |
|  | 2 doses | 0.83(0.66-1.05) | Age group & Gender |
|  | Booster dose | 0.66(0.52-0.84) | Age group & Gender |

^*^Conditional logistic regression model adjusted by age group (3-17 years, 18-64 years and over 65 years) and gender.


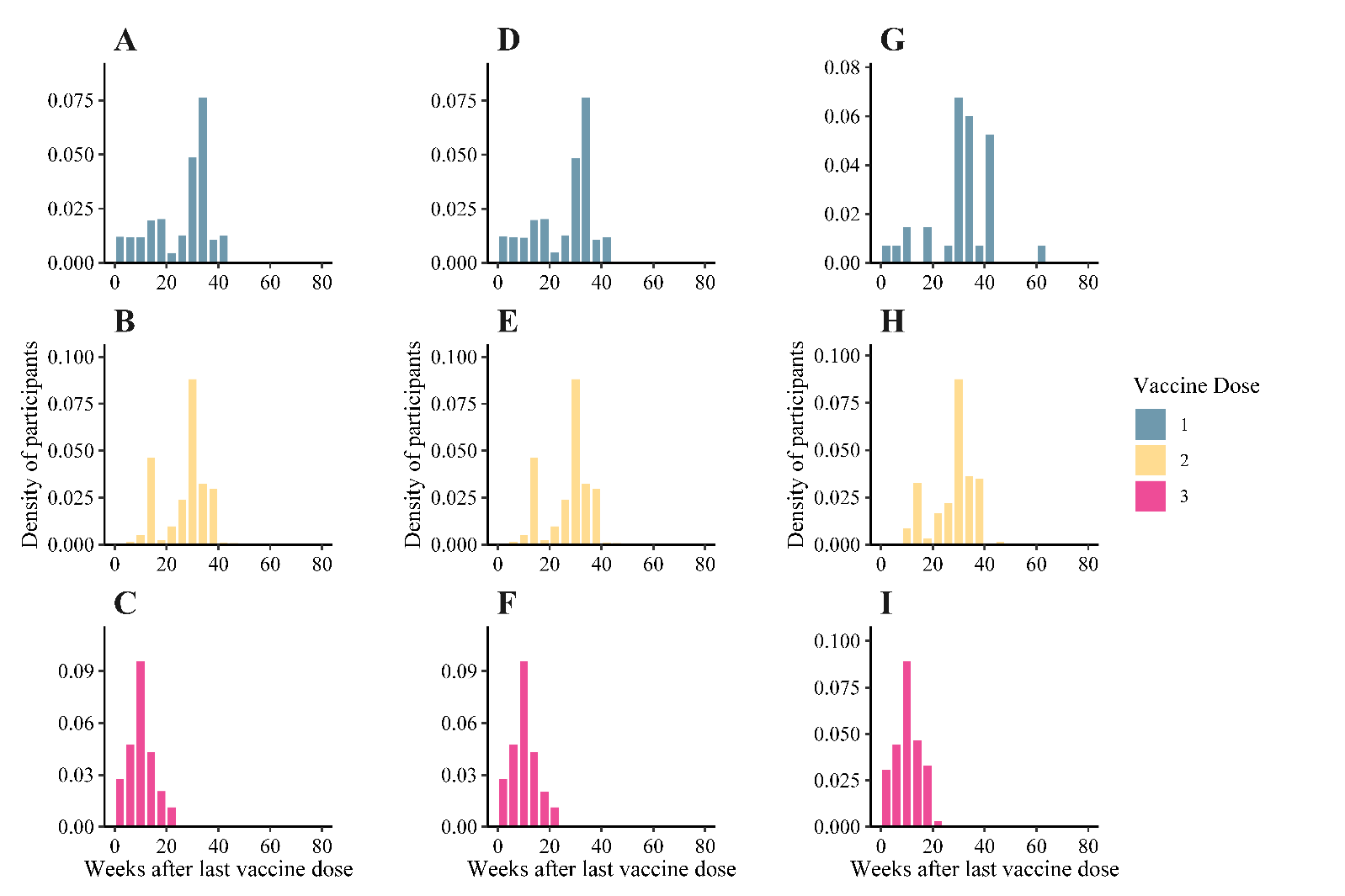


**Supplementary Figure 1**

**The distribution of the interval between the last vaccine and the date of exposure is depicted among various groups.** The distribution of interval for all participants who received 1 dose vaccine (A), 2 dose vaccine (B) and booster dose vaccine (C). The interval for control group who received 1 dose vaccine (C), 2 dose vaccine (D) and booster dose vaccine (E). The interval for the case group who received 1 dose vaccine (G), 2 dose vaccine (H) and booster dose vaccine (I).
